# Added Value of Subtraction SPECT/CT in Dual-Isotope Parathyroid Scintigraphy

**DOI:** 10.3390/diagnostics10090639

**Published:** 2020-08-27

**Authors:** Julie Wulf Christensen, Martin Krakauer

**Affiliations:** Department of Nuclear Medicine, Herlev and Gentofte Hospital, 2730 Herlev, Denmark; martin.krakauer@regionh.dk

**Keywords:** primary hyperparathyroidism, dual-isotope subtraction scintigraphy, dual-isotope subtraction SPECT/CT, Tc-99m-sestamibi SPECT/CT

## Abstract

Background: Adding subtraction single-photon emission computed tomography/computed tomography (SPECT/CT) to dual isotope (I-123 and Tc-99m-sestamibi) subtraction parathyroid scintigraphy is not widely implemented. We aimed to assess the added value of dual isotope subtraction SPECT/CT over single isotope SPECT/CT as an adjunct to dual isotope planar pinhole subtraction scintigraphy. Methods: Parathyroid scintigraphies from 106 patients with an estimated total of 415 parathyroid glands who (1) were diagnosed with primary hyperparathyroidism, (2) underwent dual isotope subtraction scintigraphy in the Department of Nuclear Medicine, Gentofte Hospital, Denmark throughout 2017 and (3) underwent subsequent parathyroidectomy, were included. The original dual isotope planar pinhole subtraction plus dual isotope subtraction SPECT/CT (dual/dual method) exams were retrospectively re-evaluated using only Tc-99m-sestamibi SPECT/CT (dual/single method). Statistics were calculated per parathyroid. Surgical results confirmed by pathology served as reference standard. Results: The dual/dual method had higher sensitivity than the dual/single method (82% (95%CI 74%–88%) vs. 69% (95%CI 60%–77%)) while specificity, positive and negative predictive values (PPV and NPV) were similar (specificity 96% vs. 93%, PPV’s 87% vs. 82% and NPV’s 89% vs. 93%). Reader confidence was higher when employing the dual/dual method (*p* = 0.001). Conclusions: The dual/dual method can be considered superior to the dual/single method in the preoperative imaging in primary hyperparathyroidism.

## 1. Introduction

Each year, 600–700 Danish patients are diagnosed with primary hyperparathyroidism (PHPT) [1,2]. PHPT is diagnosed on the basis of elevated serum calcium and elevated (or high in the normal range) parathyroid hormone (PTH) secreted from one or more hyperfunctioning parathyroid gland(s) [1,2,3]. Symptoms include musculoskeletal, cardiovascular, gastrointestinal, renal and neurological problems, and the only curative treatment is parathyroidectomy (PTx) [1,2,3,4].

Preoperative imaging is a prerequisite for minimally invasive surgery. Parathyroid dual isotope subtraction scintigraphy is a sensitive and specific preoperative imaging modality with reported sensitivities ranging between 86%–93%, specificities 98%–100% [4,5,6].

Cure rates have been found to be similar between minimally invasive surgery and bilateral neck exploration, but morbidity is higher in the latter group [7].

In our study, dual isotope (I-123 and Tc-99m-sestamibi) subtraction scintigraphy consists of early planar pinhole images and subsequent dual isotope subtraction single-photon emission computed tomography/computed tomography (SPECT/CT) with an additional delayed pinhole acquisition in case of ambiguous findings on early imaging. Though effective, processing and analyzing dual isotope subtraction SPECT/CT images is more complicated than reviewing single isotope Tc-99m-sestamibi SPECT/CT images. Thus, some nuclear medicine departments opt to use the more simple single isotope SPECT/CT.

This study aimed to evaluate the potential added information obtained from dual isotope subtraction SPECT/CT, i.e., we intended to assess and compare two methods of dual isotope subtraction scintigraphy, namely:

(1) Dual isotope subtraction pinhole imaging (I-123 and Tc-99m-sestamibi) in combination with dual isotope subtraction SPECT/CT, henceforth termed “dual/dual”.
(2) Dual isotope subtraction pinhole imaging in combination with single isotope SPECT/CT (Tc-99m-sestamibi), henceforth termed “dual/single”.

The two methods were assessed in a retrospective, blinded, head-to-head comparison in a cohort of consecutive PHPT patients who underwent post-imaging PTx.

The null hypothesis was that the two methods did not differ regarding sensitivity, specificity, or positive or negative predictive value.

## 2. Materials and Methods

### 2.1. Patients

Patients were included retrospectively. All dual isotope subtraction scintigraphies performed at the Department of Nuclear Medicine, Gentofte Hospital, Denmark from 1 January, 2017–31 December, 2017 were evaluated for inclusion. Exclusion criteria were incomplete scintigraphy (*n* = 4), post-imaging surgery outside of the Capital Region of Denmark (due to unavailability of hospital files on surgical notes) (*n* = 5), no post-imaging parathyroid surgery (*n* = 21), and previous thyroidectomy (*n* = 1)—the latter rendering subtraction scintigraphy futile.

Verifications of successful PTx were 1) a perioperative p-PTH decrease of ≥50%, 2) adenoma, hyperplasia or carcinoma confirmed on postoperative pathology and 3) normalized p-PTH and Ca^2+^ levels approximately one month postoperatively.

The study was conducted as a quality assurance study with permission from the hospital board at Herlev and Gentofte Hospital, Denmark (workzone no. 18015429, date of approval: 5 July 2018).

### 2.2. Imaging Protocol

Parathyroid scintigraphy was performed according to our standard protocol as follows: Pinhole images were performed on Philips Skylight γ-camera (Philips Healthcare, Eindhoven, The Netherlands) and SPECT/CT was performed on a Philips Precedence SPECT/CT scanner or a Siemens Symbia Intevo SPECT/CT scanner (Siemens Healthcare, Erlangen, Germany). Patients received 12 MBq (0.3 mCi) I-123 intravenously and, after 1.5 h, 600 MBq (160 mCi) Tc-99m-sestamibi. Dual isotope pinhole images were obtained after 5 min (early) and again after 2 h (late) only if early images were inconclusive. Dual isotope SPECT/CT of the neck and thorax was acquired shortly (15–30 min) after the early pinhole images.

Acquisition parameters: Energy windows were identical in planar and SPECT imaging (Tc-99m: 140 keV −7/+7; I-123: asymmetrical 159 keV −4/+10). The energy windows were chosen to minimize Tc-99m spillover to the I-123-window [8]. Pinhole image parameters: 15 min; aperture size 3 mm. SPECT imaging parameters: Dual-head, 180-degreee anterior orbit in a 90-degree angle; 128 angles, 14 s/angle; matrix 128 × 128. SPECT reconstruction used the vendor’s proprietary algorithm (Astonish^®^ or Flash3D^®^ with attenuation and scatter correction). CT was done as a non-contrast enhanced lowdose acquisition covering the neck and mediastinum and an additional diagnostic non-contrast enhanced series covering only the thyroid and immediate surroundings.

**Figure 1:**
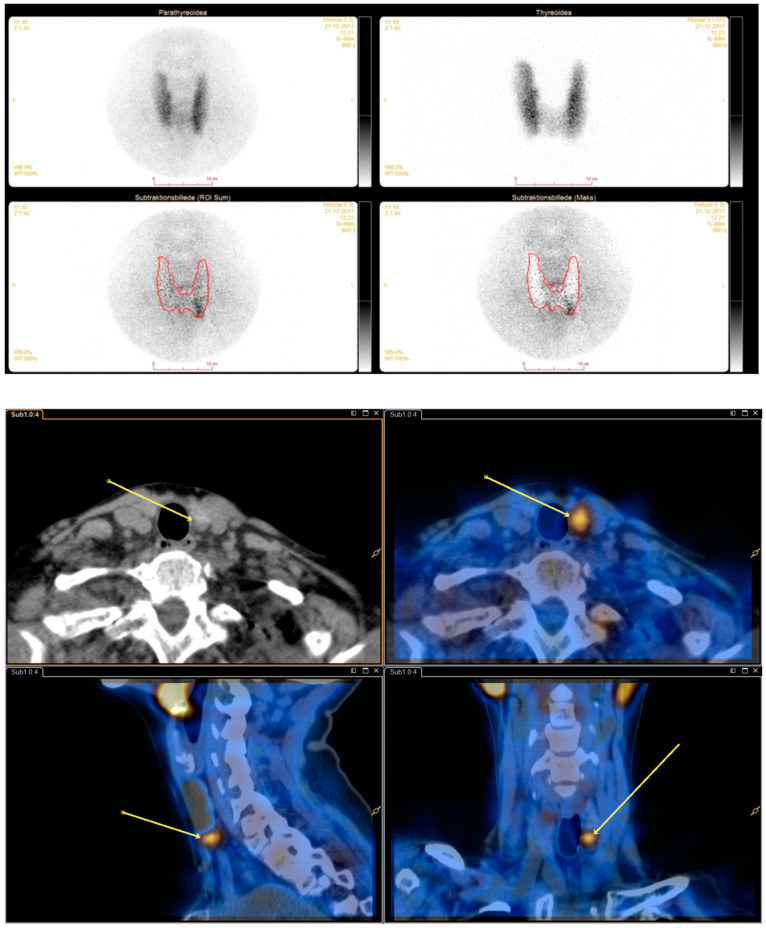
Dual isotope planar pinhole subtraction scintigraphy and dual isotope subtraction single-photon emission computed tomography/computed tomography (SPECT/CT) images. (**top image**) Upper left: Tc-99m-sestamibi pinhole. Upper right: I-123 pinhole. Middle left and right: Varying degrees of subtraction. (**bottom image**) Dual isotope subtraction SPECT/CT with and without fusion.

**Figure 2:**
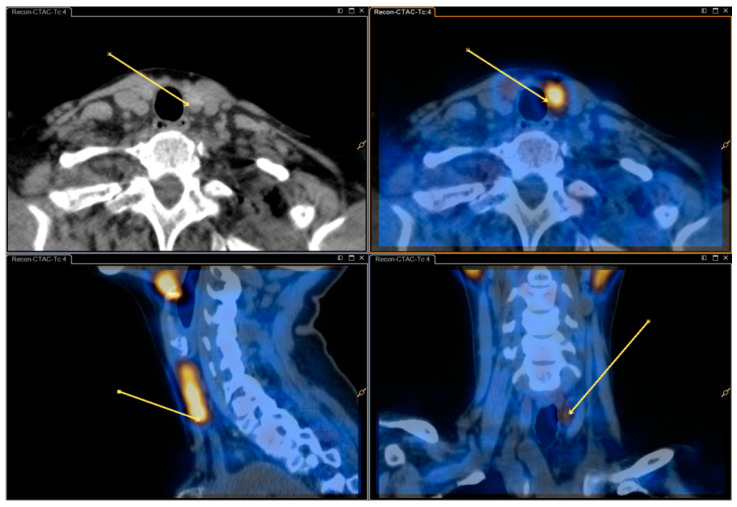
Single isotope SPECT/CT image (Tc-99m-sestamibi) with and without fusion—same patient as in Figure 1.

See Figure 1 and Figure 2 for dual/dual and dual/single images.

### 2.3. Image Analysis

#### 2.3.1. Prospective “Dual/Dual”

Scintigraphies were analyzed by a nuclear medicine specialist supervised and approved by one particularly experienced in parathyroid scintigraphy (MK).

Pinhole subtraction images were analyzed in Segami Oasis software (version 1.9, Columbia, MD, USA) where variable degrees of subtraction were available through the use of a slider until counts in the thyroid bed were equal to Tc-99m-sestamibi background counts on the neck. SPECT data were subtracted using Philips Intellispace Portal (Philips, Eindhoven, The Netherlands), the initial subtraction factor was determined by calculating the of ratio of I-123 and Tc-99m-sestamibi counts in a thyroid region of interest (ROI) in a representative coronal slice but could later be modified at the reader’s discretion.

The location of a putative hyperfunctioning parathyroid gland (HPG) was given in relation to the thyroid (left/right; upper/middle/lower third; ectopic). Confidence of the findings was graded on a three-point scale (low (1), moderate (2), high (3)).

For the purpose of this study, all image descriptions were reviewed and the number and location of HPGs as well as the confidence score were noted on a coded sheet for comparison with surgical findings.

#### 2.3.2. Retrospective “Dual/Single”

All previously processed and recorded images/reconstructions were deleted, and images subsequently anonymized to ensure re-analysis blinded to the original results.

Re-analyses were performed by an experienced nuclear medicine specialist (MK) using a similar approach to the aforementioned, except only Tc-99m-sestamibi SPECT/CT was available while the I-123 SPECT dataset and, consequently, subtraction-SPECT/CT was not.

Number and location of HPGs as well as a confidence score were recorded on a coded sheet similar to the above-mentioned.

### 2.4. Surgery and Postoperative Follow-Up

Patients underwent surgery at Department of Otorhinolaryngology, Head and Neck Surgery in The Capital Region of Denmark.

Surgery was guided by the scintigraphic findings and per-operative plasma-PTH-measurements (where a decrease of >50% indicated successful removal of the HPG(s)).

Following surgery, histological evaluation was performed to confirm the diagnosis.

After approximately one month, postoperative plasma-PTH and Ca^2+^ were determined, and normalization used to verify successful surgery.

### 2.5. Statistical Analyses

Location of each apparent HPG according to each imaging method was recorded along with confidence score of each method. Location according to surgical notes and successful surgery confirmed by perioperative PTH-decrease, postoperative pathology and postoperative PTH and Ca^2+^ served as the reference standard.

The statistical analyses were conducted using ‘R’ version 3.6.0. Vienna, Austria. URL available online: https://www.R-project.org/, accessed on 20 August 2020.Using McNemar’s Chi-square test sensitivity, specificity, positive and negative predictive values (PPV and NPV), accuracy, misclassification and Matthews Correlation Coefficient (MCC) were calculated for each modality. Results were calculated per estimated parathyroid, rather than per patient, under the assumption of four native parathyroids per patient. In order to compare the three classes of responder confidence between the two methods of analysis, we created a 3 × 3 table and used the “McNemar–Bowker test” in the statistical programme R.P values of 0.05 or less were considered statistically significant.

## 3. Results

**Figure 3:**
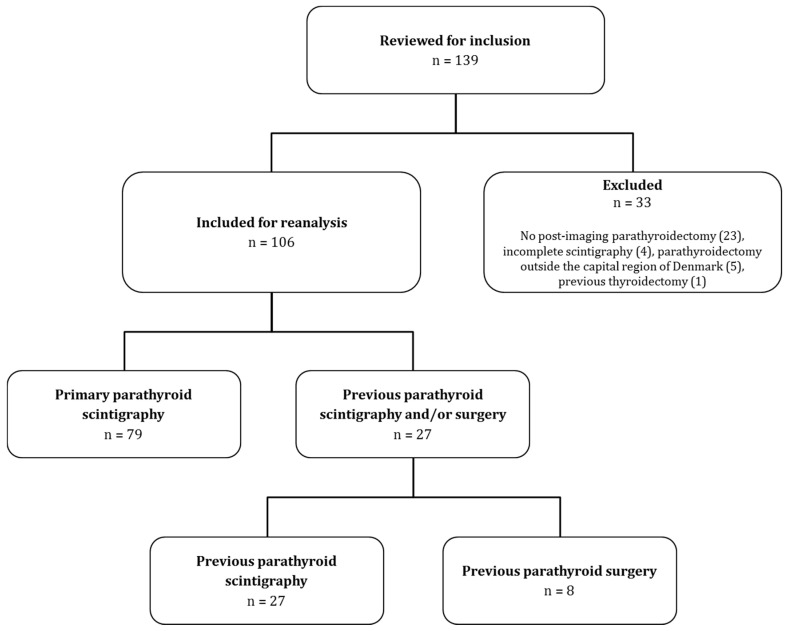
Inclusion flowchart. A total of 106 scintigraphies from between January 1, 2017 and December 31, 2017 were eligible for inclusion. Of these, 79 were primary scintigraphies and in 27 cases patients had undergone previous parathyroid scintigraphy (27) and/or parathyroid surgery (8).

Scintigraphies from 139 patients were evaluated for inclusion, and 106 patients were included for reanalysis. Of these, eight patients had previously undergone parathyroid surgery, but still had PHPT. The patients were predominantly female (84%) with a median age of 64 years (range 32–86). Twenty-seven patients had previously undergone parathyroid scintigraphy. In eight of these, scintigraphy had then been followed by parathyroid surgery. For 79 patients this was their primary parathyroid scintigraphy. See Figure 3 for inclusion flow-chart. The eight patients who had undergone previous parathyroid surgery had had nine parathyroid glands removed in total (ranging from zero to each each). Thus, assuming four native parathyroid glands per patient, the 106 patients corresponded to 415 parathyroid glands in total (i.e., (106 × 4) − 9 = 415).

The median time from scintigraphy to surgery was 101 days (range: 7–410 days). Median time from surgery to the final postoperative blood-test was 46 days (range 11–276 days). See Table 1 for baseline characteristics.

During surgery a total of 120 specimens were removed, pathology testing showed parathyroid adenoma in 93 (90 patients), parathyroid hyperplasia in 18 (13 patients) and other tissue in 9 (normal parathyroid tissue (5), normal thyroid tissue (1), lymph node (1), thymus (1) or cyst (1).

Preoperative Ca^2+^ and plasma-PTH were 1.47 mmol/L (range 1.25–1.85) and 12.15 ng/L (range 5.0–35.0), respectively. Of the 106 patients, 95 had normalized Ca^2+^ postoperatively. In five cases Ca^2+^ was continually slightly elevated, but patients were declared cured by their treating endocrinologist, in three cases Ca^2+^ was continually elevated but decreased after reoperation, and in the final two patients Ca^2+^ was continually elevated presumably due to no/insufficient removal of parathyroid tissue but watchful waiting was chosen over re-operation. A single patient never turned up for postoperative evaluation.

The average weight of the removed HPGs was 544 mg (range: 70–8000 mg).

### 3.1. Analysis

The performance of the two methods differed significantly (*p* = 0.0006). When compared to the reference standard dual/dual showed sensitivity, specificity, PPV, and NPV of 82% (95% CI 74%–88%), 93% (95% CI 90%–96%), 82% (95% CI 74%–88%), and 93% (95% CI 90%–96%), respectively. Dual/single showed a lower sensitivity of 69% (95% CI 60%–77%) but comparable specificity, PPV, and NPV of 96% (95% CI 93%–98%), 87% (95% CI 78%–92%), and 89% (95% CI 85%–92%). Accuracy and MCC were slightly higher using the dual/dual method while miss-classification ration (i.e., the inverse of accuracy) was slightly lower. See Table 2 for details.

Subgroup analyses regarding patients with and without nodular thyroid, patients with or without previous parathyroid surgery, and patients with a single HPG vs. multiglandular disease can be found online in the Supplementary Materials (not discussed here).

#### Reader Confidence

**Figure 4:**
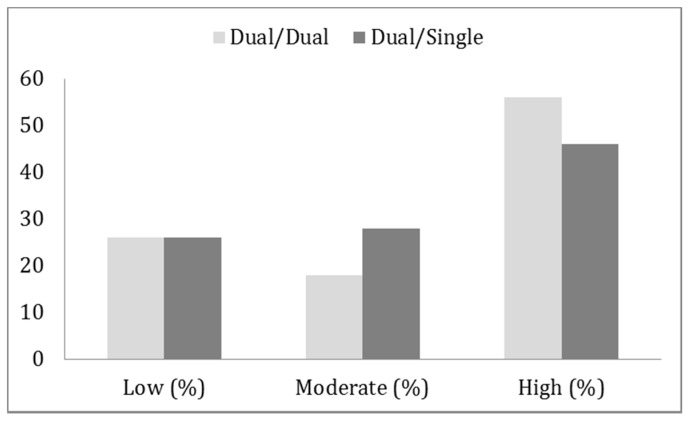
Responder confidence (low, moderate or high) according to type of imaging analysis.

Reader confidence according to type of analysis is displayed in Table 3 and graphically in Figure 4. The level of reader confidence differed significantly (*p* = 0.007) with more readings with “high confidence” with the dual/dual method.

## 4. Discussion

In a clinical context, the ability to locate HPGs preoperatively (i.e., true positives) is far more important than the ability to dismiss healthy parathyroid glands (i.e., true negatives), we find that our primary effect measure must be the sensitivities. In our study, the dual/dual method showed a higher sensitivity and thus was superior in the localization of HPGs and also yielded a higher level of reader confidence. The dual/single method proved to have slightly higher specificity.

We previously found sensitivity and specificity of 93% and 99% when using the dual/single method [4]. While the present study found a comparable specificity using the same method (96%), the sensitivity was notably lower in this new cohort (69%). This is probably due to a more heterogeneous study population in the current study. In the former study, only patients with no previous parathyroid scintigraphy or PTx were prospectively included, while all patients studied in a defined timeframe were evaluated in the present study. This included patients that had had previous off-site inconclusive imaging studies and patients with previous parathyroid/thyroid surgery thus potentially re-evaluating patients with more elusive HPGs.

Additionally, in recent years the patient population has changed due to more frequent incidentally diagnosed cases of primary hyperparathyroidism (most likely due to increased blood testing) which would in turn cause the HPGs to be smaller and more difficult to locate [9,10]. This is in agreement with the fact that in our previous study the median HPG weight was 121 mg or 22% larger than in the current study. Furthermore, differences may be contributed to the different set-ups—one a prospective study with designated readers, and one a retrospective study where the original analysis was used for statistics.

Strengths of the study were in the large number of patients compared to previous publications (i.e., 50–96 patients [4,5,6]), and the consecutive inclusion of all patients referred for imaging. A weakness was that the dual/single analyses were performed retrospectively by a single, experienced reader (MK) while the dual/dual analyses were analyzed prospectively by different readers. However, the prospective dual/dual analyses were routinely supervised by the same experienced reader in order to ensure consistency.

Several previous publications have assessed imaging setups similar to ours. A combination of subtraction pinhole and subtraction SPECT/CT (i.e., our dual/dual setup) has previously been found to have sensitivity similar to ours or higher (81%–98%) and varying specificity (67%–99%) [11,12,13]. In these studies the removed parathyroid glands were somewhat larger than in the present study (average size 700–1420 mg, compared to 544 mg in the present study) which, as mentioned, would likely increase sensitivity. Furthermore, there is a difference in imaging criteria; i.e., Asseeva et al. discriminate between “uniglandular disease”, “multiglandular disease” and “negative result” while we discriminate by the location of HPGs [11]. Due to the limited sensitivity of the dual/single method, Krčálová et al. recommend adding subtraction SPECT/CT (i.e., dual/dual) or 18F-fluorocholin-positron emission tomography / computed tomography (PET/CT) in order to increase sensitivity [14]. Others have assessed subtraction pinhole alone and/or subtraction SPECT/CT alone, the former with reported sensitivities and specificities of 75%–88% and 90% respectively, and the latter sensitivity of 86%–95% and specificity 98%–100% [6,13]. In our experience very small HPG’s may be visible only on pinhole images, therefore we do not recommend omitting planar pinhole imaging.

As mentioned, alternate protocols for preoperative imaging exist, such as ^18^F-Choline and ^11^C-Choline PET/CT. A recent review has shown that PET/CT has shown promise in the location of HPGs with sensitivities > 90% [15]. However they conclude with a need for further testing in order to confidently define the role of Choline PET/CT in preoperative diagnostics of PHPT [15].

## 5. Conclusions

Overall dual isotope subtraction SPECT/CT as an adjunct to dual isotope planar pinhole subtraction scintigraphy was more sensitive than dual isotope planar pinhole subtraction scintigraphy with Tc-99m sestamibi SPECT/CT. Moreover, dual isotope subtraction SPECT/CT resulted in higher reader confidence.

Although slightly more complicated, we do recommend applying dual isotope subtraction SPECT/CT in addition to planar pinhole subtraction imaging for preoperative parathyroid scintigraphy in patients with primary hyperthyroidism. However, the preoperative imaging strategy must always be adapted to local availability and expertise.

## Figures and Tables

**Table 1:** Baseline patient characteristics.

**Number of Patients, *n***		106	
**Gender, *n* (%)**	**Female**	89	(84%)
	Male	17	(16%)
Number of HGPs, *n* (%)	None	5	(5%)
	Single	91	(86%)
	Multiple	10	(9%)
Previous surgery, *n* (%)	Yes	8	(8%)
	No	98	(93%)
		**Median**	**Range**
Age		63.6	(32.0–86.4)
Weight of removed parathyroid (mg)		544	(70–8000)
Preoperative Ca^2+^ (mmol/L)		1.47	(1.19–1.85)
Preoperative PTH (ng/L)		15.5	(3.8–40.8)
HPGs: Hyperfunctioning parathyroid glands PTH: Parathyroid hormone			

**Table 2:** Results—Per parathyroid gland. (106 patients, 415 parathyroid glands).

	Dual/Dual		Dual/Single		
	**N**	**(%)**	**N**	**(%)**	
True positives	92	(22)	77	(19)	
True negatives	283	(68)	291	(70)	
False positives	20	(5)	12	(3)	
False negatives	20	(5)	35	(8)	
	**N**	**(95% CI)**	**N**	**(95% CI)**	**Difference**
Sensitivity	82.1	(74.0–88.1)	68.8	(59.7–76.6)	13.3
Specificity	93.4	(90.0–95.7)	96.0	(93.2–97.7)	2.6
PPV	82.1	(74.0–88.1)	86.5	(77.9–92.1)	4.4
NPV	93.4	(90.0–95.7)	89.3	(85.4–92.2)	4.1
Accuracy	90.4	(87.1–93.0)	88.7	(85.2–91.6)	1.7
Misclassification	9.6	(7.0–12.9)	11.3	(8.4–14.8)	1.7
MCC	76.1		70.6		5.5

The number of true positives, true negatives, false positives and false negatives as well as sensitivity, specificity, positive and negative predictive values (PPV and NPV), Matthews Correlation Coefficient (MCC) and miss-classification. Each modality is compared to the reference standard (i.e., surgery confirmed by pathology and clinical follow up). *p* = 0.0006 (When comparing dual/dual to dual/single).

**Table 3:** Scintigraphy “responder confidence” per parathyroid. N = number of hyperfunctioning parathyroid glands found using each method.

	N	Low N (%)	Moderate N (%)	High N (%)
Dual/dual	111	29 (26%)	20 (18%)	62 (56%)
Dual/single	89	23 (26%)	25 (28%)	41 (46%)

Dual/dual: Dual isotope planar pinhole subtraction and SPECT/CT images (I-123 and Tc-99m-sestamibi).Dual/single: Dual isotope planar pinhole subtraction (I-123 and Tc-99m-sestamibi) and single isotope SPECT/CT images (Tc-99m-sestamibi).

## Supplementary Materials

The following are available online at https://www.mdpi.com/2075-4418/10/9/639/s1.
