# Supplementary material for: Added Value of Subtraction SPECT/CT in Dual-Isotope Parathyroid Scintigraphy
Source: Diagnostics (Basel). 2020 Aug 27;10(9):639. doi: 10.3390/diagnostics10090639 (PMC7555464; doi:10.3390/diagnostics10090639)
Supplement: Supplementary file 1 [file diagnostics-10-00639-s001.pdf]

diagnostics

Supplementary Materials: The following are available online at www.mdpi.com/xxx/s1:

Table 4. Results – subcategories per parathyroid gland (106 patients, 415 parathyroid glands)

	True pos N (%)	True neg N (%)	False pos N (%)	False neg N (%)	Sensitivity (95% CI)	Specificity (95% CI)	PPV (95% CI)	NPV (95% CI)	P
**Nodular thyroid (N = 147)**									
Dual / dual	33 (22%)	97 (66%)	8 (5%)	9 (6%)	78.6 (64.1-88.3)	92.4 (85.7-96.1)	80.5 (66.0-89.8)	91.5 (84.7-95.5)	0.055
Dual / single	26 (18%)	100 (68%)	5 (3%)	16 (11%)	61.9 (46.8-75.0)	95.2 (89.3-98.0)	83.9 (67.4-92.9)	86.2 (78.8-91.3)	
Difference					16.7	2.8	3.4	5.3	
**Non-nodular thyroid (N = 268)**									
Dual / dual	59 (22%)	186 (69%)	12 (4%)	11 (4%)	84.3 (74.0-91.0)	93.9 (89.7-96.5)	83.1 (72.7-90.1)	94.4 (90.3-96.9)	0.006
Dual / single	51 (19%)	191 (71%)	7 (3%)	19 (7%)	72.9 (61.5-81.9)	96.5 (92.9-98.3)	87.9 (77.1-94.0)	91.0 (86.3-94.1)	
Difference					11.4	2.6	4.8	3.4	
**Previous parathyroid surgery (N = 23)**									
Dual / dual	8 (35%)	14 (61%)	1 (4%)	0 (0%)	100 (67.6-100)	93.3 (70.2-98.8)	88.9 (56.5-98.0)	100 (78.5-100)	0.371
Dual / single	5 (22%)	14 (61%)	1 (4%)	3 (13%)	62.5 (30.6-86.3)	93.3 (70.2-98.8)	83.3 (43.7-97.0)	82.4 (59.0-93.8)	
Difference					37.5	0	5.6	17.6	
**No previous parathyroid surgery (N = 392)**									
Dual / dual	84 (21%)	270 (69%)	19 (5%)	19 (5%)	81.6 (72.7-88.5)	93.4 (89.9 – 96.0)	81.6 (73.9 – 87.3)	93.4 (90.4 – 95.5)	0.002
Dual / single	72 (18%)	278 (71%)	11 (3%)	31 (8%)	69.9 (60.1 – 78.6)	96.2 (93.3 – 98.1)	86.8 (78.3 – 92.2)	90.0 (87.0 – 92.3)	

Difference					11.7	2.8	5.2	3.4	
**Single HPG (N = 359)**									
Dual / dual	78 (22%)	251 (70%)	16 (4%)	14 (4%)	84.8 (75.8-91.4)	94.0 (90.45-96.54)	83.0 (75.1-88.8)	94.7 (91.7-96.7)	0.005
Dual / single	68 (19%)	258 (72%)	9 (3%)	24 (7%)	72.9 (63.7-82.5)	96.6 (93.70-98.45)	88.3 (79.7-93.6)	91.5 (88.4-93.8)	
Difference					11.9	2.6	5.3	3.2	
**Multiple HPGs (N = 39)**									
Dual / dual	14 (36%)	19 (49%)	1 (3%)	5 (13%)	73.7 (48.8-90.9)	95.0 (75.1-99.9)	93.3 (67.0-99.0)	79.1 (64.0-89.0)	0.041
Dual / single	9 (23%)	20 (51%)	0 (0%)	10 (26%)	47.4 (24.5-71.1)	100 (83.2-100)	100	66.7 (56.6-75.4)	
Difference					26.3	5.0	6.7	12.4	

Number of true positives, true negatives, false positives and false negatives as well as sensitivity, specificity and positive and negative predictive values (PPV and NPV). Each modality is compared to the gold standard (i.e. surgery confirmed by pathology testing).
Subgroup analysis.
P values when comparing *dual/dual* to *dual/single* using McNemars test within each category.

© 2020 by the authors. Submitted for possible open access publication under the terms and conditions of the Creative Commons Attribution (CC BY) license (http://creativecommons.org/licenses/by/4.0/).
